# Spirulina/Arthrospira/Limnospira—Three Names of the Single Organism

**DOI:** 10.3390/foods13172762

**Published:** 2024-08-30

**Authors:** Maria A. Sinetova, Elena V. Kupriyanova, Dmitry A. Los

**Affiliations:** K.A. Timiryazev Institute of Plant Physiology, Russian Academy of Sciences, 127276 Moscow, Russia; maria.sinetova@mail.ru (M.A.S.); ivlaanov@mail.ru (E.V.K.)

**Keywords:** *Arthrospira*, *Limnospira*, *Spirulina*, bioactive substances, biomass, biotechnology, cyanobacteria, natural pharmaceuticals

## Abstract

Recent advances in research techniques have enabled rapid progress in the study of spirulina, an ancient edible cyanobacteria. Nowadays, spirulina species are classified into three genera: *Spirulina*, *Arthrospira*, and *Limnospira*. The latter now refers to industrially manufactured spirulina strains. Whole-genome sequencing revealed gene clusters involved in metabolite production, and the physiology of spirulina. Omics technologies demonstrated the absence of hazardous compounds in spirulina cells, confirming the safety of this biomass as a food product. Spirulina is a good source of different chemicals used in food manufacturing, food supplements, and pharmaceuticals. Spirulina’s enrichment with inherent biologically active substances makes it a potential supplier of natural products for dietary and pharmaceutical applications. Spirulina is also a prospective component of both terrestrial and space-based life support systems. Here, we review current breakthroughs in spirulina research and clarify fallacies that can be found in both professional literature and public media.

## 1. A Historical and Taxonomic Introduction: From *Spirulina* and *Arthrospira* to *Limnospira*

*Spirulina* (*Arthrospira* or *Limnospira*) belongs to the phylum Cyanobacteria (previously known as blue-green algae), which is one of the ancient types of living organisms on Earth [1]. Cyanobacteria, like other prokaryotes, do not have a nucleus, while algae all have a nucleus and belong to Eukaryotic kingdoms. *Spirulina*, although a multicellular organism (filamentous cyanobacterium), has no nucleus and no other cellular organelles. *Spirulina* cells are packed with photosynthetic thylakoid membranes and surrounded by the cytoplasmic and outer membranes. They carry a single copy of the circular chromosome organized in a prokaryotic-type nucleoid. Studies of fossil microorganisms in Precambrian rocks (3.5–0.5 billion years ago) revealed cyanobacterial communities [2,3]. Multiple species of modern cyanobacteria can be found in practically any environment, from soil to freshwater and seawater, as well as extreme ecosystems such as hot springs, soda and salt lakes. It is accepted that cyanobacteria are very conservative and have changed insignificantly morphologically and, most likely, physiologically over the last at least 2 billion years [1,4].

*Spirulina* (*Arthrospira* or *Limnospira*) was used as a food source by the Aztecs and other Mesoamericans until the 16th century. A Spanish chronicle describes harvesting *Spirulina* from Lake Texcoco, Mexico, in 1521 [5,6] and selling it in the form of blue-green yummy cakes called “tecuitlatl” [7]. In 1827, the main constituent of *tecuitlatl* had been identified and described by botanists as *Spirulina oscillarioide*, a spiral multicellular cyanobacteria (at that time, blue-green algae) [8]. In 1852, *Spirulina* was renamed *Arthrospira* due to some morphological reasons (arthro from Greek άρθρωση (árthrosi)—joint and spira—σπιράλ (spiral) [9]. In 1931, *Spirulina*/*Arthrospira* was re-discovered as a dominant phytoplankton in lakes of East Africa [10]. In 1932, the class Cyanophyceae was revised again, the genus *Arthrospira* was invalidated, and all regularly helically coiled oscillatorian organisms were placed in the previously described [8] genus *Spirulina* [11,12].

In 1940, *Spirulina* was reintroduced to the world from a sample collected from a cake called “dihé” produced near Lake Chad in Africa [13]. In the 1960s, a trans-Saharan expedition stumbled upon dihé and *Spirulina* again. Dihé samples appeared to be dried purees mostly consisting of filamentous cyanobacteria *Spirulina platensis* [14]. At about the same time, but on the other side of the globe, *Spirulina*/*Arthrospira* was found again in the waters of Lake Texcoco, Mexico. Unsurprisingly, there it had been used as food by the natives living in the area [15]. It became clear that these two different cultures, living far apart, discovered the nutritional value of *Spirulina* independently. Finally, both cakes, tecuitlatl and dihé, appeared mostly identical and supposedly consisted of two species, *Spirulina maxima* and *Spirulina platensis*, respectively [12,16].

For a long time, most authors followed Geitler’s classification [11]; however, the question turned out not to be as simple as it appeared in 1932. Many researchers realized that genera *Spirulina* and *Arthrospira* are distinct and returned to using two names. The supporting evidence can be summarized as follows:

1. Trichome helix usually nearly closed (i.e., spring-like coil); cross walls thin and usually invisible with light microscope; cell width typically 2–4 µm, permanently motile by rotation. Form—genus *Spirulina*.
2. Trichome helix usually open (i.e., as stretched spring); cross walls visible with light microscopy; cell width typically 6–12 µm; gas vesicles generally present. Form—genus *Arthrospira* [17].

Many species classified as *Spirulina*, including those grown commercially and sold as *Spirulina*, have had to be reclassified as *Arthrospira*. *Spirulina*, however, is now so widely known that it appears inevitable that the term will endure; however, it should be written as spirulina, with no capital letter and no italics [18]. Yet, it should be considered that both *Arthrospira* and *Spirulina* species are present in modern taxonomy. Moreover, *Arthrospira* has been recently divided into two distinct groups (see below).

*Arthrospira* includes 23 approved species which form mats or live as single trichomes. Three species are marine, three are from environments with high pH, and the others are freshwater species. The species have been found across all continents and both poles, the Arctic and Antarctica. In warmer places, planktonic varieties tend to produce large blooms, which are historically collected by natives for direct consumption as food.

That would be an adequate ending to this long classification story. However, the pirouettes of *Spirulina*/*Arthrospira* taxonomy are not over. *Arthrospira* is one of nineteen genera in the Microcoleaceae family, which belongs to the Oscillatoriales order [19]. Several studies have focused on the evolutionary relationship of *Arthrospira* to taxa other than the type species, *Arthrospira jenneri* [9,20]. Unlike more popular cultivated species, *A. jenneri* (first mentioned in 1845 as *Spirillum jenneri*) came from freshwaters with low salinity [21], while the commercial strains identified as *A. maxima*, *A. fusiformis*, and *A. platensis* have been isolated mostly from highly alkaline environments in tropical and subtropical regions. These facts together with the absence of information about the type species wrongly implied that a preference for high pH and salinity is a common property shared by the whole genus [22].

**Figure 1:**
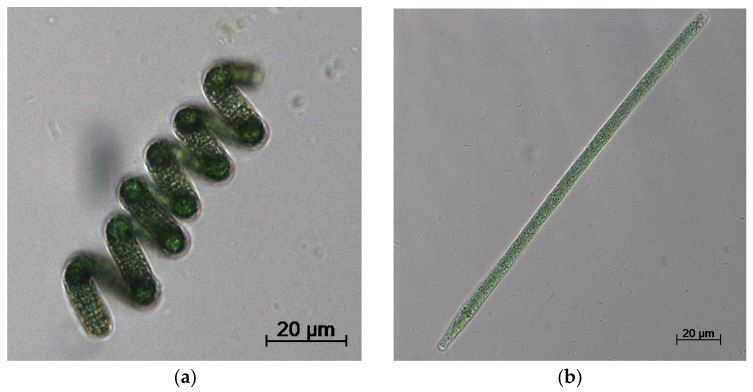
Microscopic visualization of spirulina. Spiral *Limnospira* sp. IPPAS B-1526 (**a**) and linear *Limnospira* sp. IPPAS B-287 (**b**) from the collection of microalgae and cyanobacteria of K.A. Timiryazev Institute of Plant Physiology, Russian Academy of Sciences (IPPAS, Moscow, Russia).

In 2019, the commercialized *Arthrospira*/*Spirlina* species (more than 30 varieties) were analyzed using molecular (16S rRNA, 16S-23S rRNA ITS, and *cpcBA*-IGS sequences) and phenotypic (13 morphological and morphometric characteristics) data. On this basis, *Arthrospira* strains were divided into three clusters or taxa. The main two taxa, Taxon I and Taxon II, included planktonic representatives, while Taxon III comprised the benthic cyanobacteria [23]. Although some reports based on the 16S rRNA gene and *cpcBA*-IGS sequences still consider *Arthrospira* taxa as monophyletic [24], the analysis of 33 new strains of *Arthrospira* isolated from plankton samples collected in Mexico, East Africa, and India also divided them into two groups, Mexican and African/Indian clusters, respectively [25]. Figure 1 depicts two nearly genetically identical species with completely different morphological characteristics.

The modern polyphasic approach and developments in DNA sequencing techniques somehow simplified the challenges of taxonomic issues and allowed more accurate phylogenetic analysis of cyanobacterial species [20]. Further studies of *Arthrospira* species revealed severe differences between the type strain *A. jenneri* and commercially produced strains [26]. Finally, at this moment, the name *Limnospira* is proposed for economically important species that include formally known *A. maxima*, *A. fusiformis*, and *A. platensis* [26]. In other words, the up-to scientific name of spirulina that one can buy on the market is *Limnospira* spp. [18]. There is quite a funny explanation for the term “spirulina” on Wikipedia: “…spirulina refers to the dried biomass of *A. platensis*” (https://en.wikipedia.org/wiki/Spirulina_(dietary_supplement) accessed on 13 August 2024). We consider that “dry” and “wet” biomass, as well as living cells of *Arthrospira*/*Spirulina*/*Limnospira*, all may be covered by the term spirulina.

Summarizing the taxonomical pirouettes of spirulina, one can conclude that three groups of organisms are distinguished at present:

1. *Spirulina* spp.—12 species with the type strain *Spirulina subsalsa*;
2. *Arthrospira* spp.—about 35 species with the type strain *Arthrospira jenneri*;
3. *Limnospira* spp.—4 species with the type strain *Limnospira fusiformis* (formerly *Spirulina fusiformis*), as well as *Limnospira* (formerly *Spirulina* or *Arthrospira*) *platensis*, *Limnospira* (formerly *Spirulina* or *Arthrospira*) *maxima*, and *Limnospira* (formerly *Spirulina* or *Arthrospira*) *indica*, which are commonly mass-cultivated to produce spirulina biomass for sale on the market.

**Figure 2:**
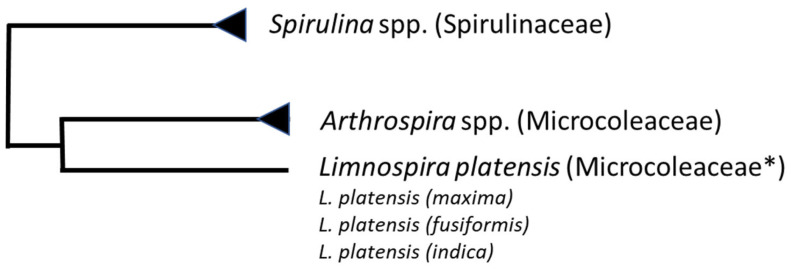
A simplified scheme of taxonomic positioning of *Spirulina*, *Arthrospira*, and *Limnospira* families according to Algabase (https://www.algaebase.org/search/species/ (accessed on 14 August 2024 [29])). * According to NCBI taxonomy browser, all *Limnospira* substrains belong to Sirenicapillariaceae family [30].

When *Limnospira* spp. was separated from *Arthrospira* in 2019, there was doubt about *A. platensis*; it was not definitely assigned to the *Limnospira* clade [26]. Recent advances in whole-genome sequencing allowed the comparison of complete and draft genomes of *Limnospira* strains (30 genomes of strains in total, many of which have been newly isolated) [27]. This comparison revealed that the genus *Limnospira* is rather monospecific and represented by *L. platensis* PCC 7345 (number in Pasteur Culture Collection; accessed on 14 August 2024) as a reference strain. All other strains, including the previously named *L. fusiformis*, *L. indica*, *L. maxima*, and all other related strains should be considered as substrains of *L. platensis* [27], similarly to *Synechocystis* sp. PCC 6803 substrains of the model unicellular cyanobacterium [28]. A current scheme of taxonomic relationships among the *Spirulina*, *Arthrospira*, and *Limnospira* families is depicted in Figure 2.

The latter finding means that the authorization for food applications may be extended to all existing and future strains of *Limnospira*. This greatly simplifies the procedure of the commercialization of *Limnospira* strains for human consumption regulated by the European Commission (in the EU), which publishes the Novel Food status Catalogue (NFC; accessed on 14 August 2024).

## 2. Biochemical Characterization of Spirulina

In general, *Limnospira* (spirulina) biomass contains 60–70% protein, 15–20% carbohydrates, 5–8% lipids, 6–8% minerals (K, Na, Ca, Fe, Mg, and Mn) (dry *w*/*w*) [31]. It also contains pigments chlorophyll *a*, zeaxanthin and canthaxanthin, vitamins B1, B2, B3, B6, B9, B12, vitamin C and E, and colored proteins c-phycocyanin and allophycocyanin [32]. Spirulina resists various concentration of salt, for example, NaCl at concentrations ranging from 5 to 40 g L^−1^ does not affect absolute or relative content of proteins (including phycocyanins), lipids, FAs, carbohydrates, chlorophyll, carotenoids [33]. Spirulina may be cultured at temperatures of 30 ± 5 °C without significant changes in growth or in the above-mentioned parameters.

### 2.1. Protein Value of Spirulina

Spirulina is one of the richest protein sources of microbial origin (460–630 g protein per kg biomass on a dry weight basis in *L. platensis* and 600–710 g kg^−1^ in *L. maxima*) [34]. These values are comparable with meat (710–760 g kg^−1^), soybeans (~400 g kg^−1^), and *Chlorella vulgaris* (510–530 g kg^−1^) [35]. The advertisements report that spirulina has 670% more surplus protein than tofu (bean curd), 5100% more radical iron than spinach, 3100% β-carotene compared to carrots, 180% Ca^2+^ compared to total milk. Spirulina is a protein-rich cyanobacterium (60–70% *w*/*w*) with a balanced amino acid composition and a prominent source of c-phycocyanin (47% of total protein)—a blue pigment–protein complex used as a natural blue food colorant [36]. C-phycocyanin is also used in pharmaceutical industries and as a functional food due to its antioxidant, neuroprotective, anti-inflammatory, and hepatoprotective properties [37]. It can also be employed as a fluorescent probe for cellular and molecular detection [38].

Nutritional composition of spirulina powder and the protein quality, in terms of amino acid type content, varies depending on the manufacturer [39]. Total protein content varies from 0.55 to 0.7 g g^−1^ of dry powder. The essential amino acids are represented by (mg g^−1^) leucine (30–56), tryptophan (10–80), methionine (14–60), phenylalanine (26–38), lysine (30–60), threonine (15–33), isoleucine (30–40), histidine (10–15), and valine (30–45). Non-essential amino acids are alanine (40–50), arginine (30–50), aspartic acid (15–60), cysteine (60–70), glutamic acid (60–90), glutamine (80), glycine (20–40), proline (20–30), serine (28–45), and tyrosine (20–30) mg g^−1^ [39,40,41]. According to these results, the tested spirulina biomass is a good source of essential and non-essential amino acids.

### 2.2. Lipids and Fatty Acids

Cyanobacterial membranes include the cytoplasmic (plasma) membrane and the thylakoid membranes. Both types of membranes contain four major glycerolipids: monogalactosyldiacylglycerol (MGDG), digalactosyldiacylglycerol (DGDG), sulfoquinovosyldiacylglycerol (SQDG), and phosphatidylglycerol (PG). The molecular motion of these glycerolipids is mostly determined by the degree of unsaturation of the fatty acids esterified to the glycerol backbones [42]. The activity of fatty acid desaturases, enzymes that add double bonds into specified locations of fatty-acyl chains in lipids, determines the level of unsaturation [43]. Changes in FA unsaturation affect multiple functions of membrane-bound proteins, such as the photochemical and electron transport reactions that occur in the thylakoid and cytoplasmic membranes of cyanobacterial cells [44].

The fatty acid (FA) composition of cyanobacterial lipids is determined by their chain length (number of carbon atoms) and the amount of double bonds. The FA chain length in cyanobacteria typically ranges from C14 to C18. The amount of double bonds in these chains can range from 0 to 4, resulting in totally saturated FAs (no double bonds), monoenoic (one double bond), dienoic (two double bonds), trienoic, and tetraenoic (three and four double bonds, respectively) FAs. The FA composition of each cyanobacterial species is so preserved that it can be utilized as a phylogenetic marker [45,46,47].

Christine Kenyon established the classification of cyanobacteria based on their FA content [48,49], which was later modified by Murata and coworkers [50]. The most recent classification of cyanobacteria based on the number of double bonds in FAs [51] divides all cyanobacterial strains into four distinct groups. Group 1 organisms form a single double bond at the ∆^9^ position of fatty acids (typically C16 or C18), which are esterified at the *sn*-l position of the glycerol molecule. In Group 2 cyanobacteria, C16 palmitic and C18 stearic acids are desaturated at the ∆^9^ and ∆^12^ positions. In Group 3, C18 acid is desaturated at ∆^9^, ∆^12^, and at either the ∆^6^ or ∆^15^(ω^3^) positions. Finally, in Group 4, the C18 stearic acid is desaturated at the ∆^6^, ∆^9^, ∆^12^, and ∆^15^ (ω^3^) positions.

**Figure 3:**
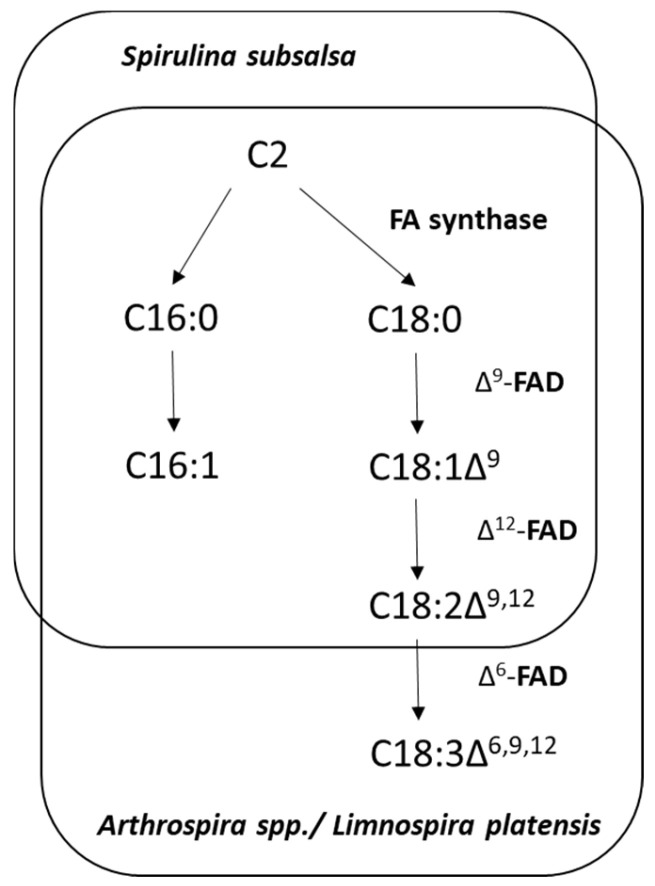
A scheme of fatty acid (FA) synthesis and desaturation in *Spirulina subsalsa* and *Arthrospira*/*Limnospira* species. One complex FA synthase is responsible for cyclic elongation of malonyl-CoA (C2) to palmitoleate (C16:0) and stearate (C18:0). These two FAs undergo desaturation (the formation of double bonds). Palmitic acid, C16:0, is desaturated into monoenoic palmitoleic acid C16:1∆^9^ by ∆^9^-FAD. Stearic acid, C18:0, is desaturated into monoenoic oleic acid, C18:1∆^9^, by the same ∆^9^-FAD. Oleic acid is further desaturated into dienoic linoleic acid, C18:1∆^9,12^, by ∆^12^-FAD, and to trienoic γ-linolenic acid, C18:1∆^6,9,12^, by ∆^6^-FAD. The chromosome of spirulina contains three genes for FADs mentioned above, each only in one copy. No FA elongases exist in spirulina for building FA chains longer that C18.

According to this classification, *Arthrospira* and *Limnospira* species belong to Group 3, Subgroup 3γ. Those strains have Δ6-, Δ9-, and Δ12-fatty acid desaturases (FADs), which produce trienoic γ-linolenic acid (GLA—18:3∆^6,9,12^) as a final product of FA desaturation [52] (Figure 3). Cyanobacterial lipids include a relatively small variety of unsaturated FAs (UFAs), specifically C14-C18 FAs with one to four double bonds [51,53]. Some reports on FAs longer than C18 in cyanobacteria, including spirulina [54,55,56], most likely, describe contamination by fungi/lichens, microalgae, or something else. Cyanobacterial genomes lack FA elongase genes, which ensure C2 elongation of C18 into C20 and further into C22. FA elongases are only found in the endoplasmic reticulum of eukaryotic cells. The attempts to express these genes in cyanobacterial cells were unsuccessful (our unpublished observations).

Currently, commercial GLA is derived largely from plant seed oils: evening-primrose oil (*Oenothera biennis*) (~10% of total FAs) [57], blackcurrant seed oil (*Ribes nigrum*) (up to 20%) [58,59], and borage seed oil (*Borrago officinalis*) (20–25%) [60]. It is believed to be the most expensive edible oil. Ciferri [16] and Roughan [61] were the first to indicate that spirulina may be used as the richest source of dietary GLA. The fatty acid composition and GLA content of 19 different spirulina strains were studied at various temperatures, light intensities, and growth phases [62]. The study found that spirulina has a GLA content of around 32% FAs and 1.5% biomass (dry weight). It should be noted that whereas 18 of the 19 spirulina strains contained GLA, no GLA was detected in *S. subsalsa*, which had a very high content of palmitoleic acid [62,63]. Considering that FA composition of cyanobacteria is a phylogenetic marker [44,45,46,47], we would venture to say that the examined strain (*S. subsalsa*) is a true *Spirulina* strain that does not belong to the *Arthrospira*/*Limnospira* clade. At least, the genomes of *S. subsalsa* listed in the NCBI database do not contain any genes homologous to Δ6-FAD. Therefore, true Spirulina species should be attributed to Group 2 cyanobacteria, according to FA-based classification [50,51].

The maximum GLA content was recorded in most strains at 30–35 °C. High light intensities at high temperatures (38 °C) had a significant effect on FA content, lowering it by up to 46%. A week of darkness resulted in a 50% increase in cellular GLA concentration in spirulina cultures [64]. GLA content in spirulina can also be increased by cultivating it in light–dark cycles [65] or using urea as a nitrogen source [66]. GLA accounts for 10–20% of all FAs in *L. maxima* and up to 50% in *L. platensis*.

Reports and advertisements claiming the presence of ω3 FAs in spirulina [56,67] are either speculative or based on limited evidence. Natural spirulina strains lack any ω3-FAD genes in their genomes. However, genetic engineering may occasionally introduce it in the future. It should also be noted that natural cyanobacteria strains do not accumulate storage lipids, such as triacylglycerides (TAGs), and thereby do not produce (much) plant-like oils. Theoretically, the latter can also be engineered and developed. TAG-like molecules (lipid X) have been detected in picomol concentrations in the model *Synechocystis* [68,69]. Later, lipid X was defined as plastoquinone-C esterified with 16:0 or 18:0 by the Slr2103 acyltransferase [70].

Table 1 summarized the appearance of commonly known biochemical compounds in spirulina to avoid confusion that arises due to some published sources.

### 2.3. Phenolic Compounds

Flavonoids, phenolic acids, and tannins are thought to be the most important contributors to plants’ antioxidant properties. Cyanobacteria have also been identified as producers of many biologically active secondary metabolites with potential therapeutic capabilities, including anti-proliferative, anti-inflammatory, and anti-infective effects. The examination of phenolic components in 20 terrestrial cyanobacteria strains revealed varying amounts of polyphenolics: gallic, chlorogenic, caffeic, vanillic, and ferulic acids, as well as flavonoids rutin, quercetin, and kaempferol [74]. Filamentous cyanobacteria have been found to contain quinic acid, catechin, epicatechin, and apiin with concentrations ranging from 14.86 to 701.69 μg g^−1^ dry weight [75]. In general, filamentous (multicellular) cyanobacterial species have a greater diversity of phenolic chemicals than unicellular species [76].

In spirulina, total phenolic compounds account for 4.5–17 mg g^−1^ (dry weight), depending on culture conditions, and flavonoids, 1.32–5.12 mg g^−1^. The HPLC profiling of phenolic extracts of spirulina revealed a number of phenolic acids and flavonoids: gallic, chlorogenic, cinnamic, *p*-OH-benzoic acids, and pinostrobin (5-hydroxy-7-methoxyflavanone) [77]. The major phenolics reported in spirulina were acacetin (5,7-dihydroxy-4″-methoxyflavone) (53.62%) and pinocembrin (5,7-dihydroxyflavanone) (41.28%) [78]. Other reports point to rutin (1.4 mg g^−1^), naringenin (0.4 mg g^−1^), quercetin (0.3 mg g^−1^), kaempferol, and quercetin-3-β-glycoside, as well as neochlorogenic acid at ~2 mg g^−1^ for lyophilized and ~60 mg g^−1^ for convection-dried spirulina [79]. Thus, spirulina may be regarded as a valuable source of phenolics with strong antioxidant activity, and it may be used as a natural antioxidant.

### 2.4. Polysaccharides

So far, over 83 different polysaccharides have been isolated from the spirulina genus. Their physical and chemical properties, such as glycosyl sequences and linkage types, branch structure, and chain conformation were characterized [80]. Early investigations suggested that spirulina polysaccharides are composed of glucan [81]. Later on, it was determined that the majority of spirulina heteropolysaccharides are mainly composed of glucose, rhamnose, xylose, mannose, galactose, arabinose, and fucose with different molar ratios, while ribose, fructose, galacturonic acid, and glucuronic acid are relatively rare [82,83]. Polysaccharides are known as spirulina’s key bioactive components. Their documented bioactivities appear to cover all fields of known pharmacological activities, including regulation of intestinal microflora, as well as organ-protective and neuroprotective, immunomodulatory, antitumor, antioxidant, antithrombotic, antiatherosclerotic, hematopoietic, antidiabetic, antiviral, antibacterial, antiradiation, antiaging, antifatigue, anti-anoxic, antiangiogenic, anti-inflammatory, and wound healing effects (for reviews, please see [80,84,85,86]).

Spirulina metabolites (extracts) have been utilized for centuries as protein- and vitamin-rich health food supplements or nutraceuticals. As previously stated, they exhibit antioxidant and antiviral (HIV, HSV, and perhaps SARS-CoV) effects [87], and the ability to stimulate the immune system. Most of the specific active components in those extracts have not been characterized. It is yet unclear whether the therapeutic effects of spirulina are related with specific molecules that can be extracted and defined, or if this is a synergistic impact of numerous metabolites (which remain to be isolated, identified, and mixed in optimal quantities).

Calcium spirulan (Ca-SP), a sulfated polysaccharide of spirulina, should be highlighted among a few described substances. Ca-spirulan consists of rhamnose, 3-O-methyl-rhamnose, 2,3-di-O-methyl-rhamnose, 3-O-methylxylose, uronic acids, sulfate groups, and calcium ions chelated with sulfate groups [88]. Spirulan specifically inhibits the penetration and replication of enveloped viruses, including Herpes simplex viruses type 1 and 2 (HSV-1 and HSV-2), human cytomegalovirus (HCMV), measles, mumps, influenza A, and HIV-1 [89,90].

## 3. Culturing Spirulina: Quality Control and Standardization Issues

Spirulina quality varies depending on producer and distributor. There may be considerable differences across batches made by the same manufacturer. Spirulina is currently produced in at least 25 countries, including Benin, Brazil, Burkina Faso, Chad, Chile, China, Costa Rica, Cote d’Ivoire, Cuba, Ecuador, France, India, Israel, Japan, Madagascar, Mexico, Myanmar, Peru, Russia, Spain, Tanzania (Zanzibar), Thailand, Togo, the United States of America, and Vietnam. The total yearly commercial output of spirulina is around 10,000 tons (dry biomass), with China producing roughly half of that [91].

Spirulina is mass-produced in outdoor ponds with direct sunlight, in closed greenhouses, and in closed photobioreactors (PBRs). Sunlight under the open pond means “cheap and dirty”, while closed installations with artificial lighting mean “clean but expensive”. PBRs use artificial lighting and air–CO_2_ mixtures to accelerate the growth of cyanobacterial cultures. Of course, the latter raises the cost of biomass production, but it can guarantee the quality of the end product. Spirulina powder supplied on the market is primarily manufactured outdoors. It is considered that in general, such products have no apparent negative impact on the human body, reproductive performance, embryo/fetus development, or growth. Nonetheless, in cultures without control for contamination, spirulina biomass may contain low quantities of mercury and other heavy metals that constitute a direct health risk [92]. Spirulina powder or tablets produced from the materials collected in open ponds may contain rodent hair, bird feathers, and excrement as well as many other interesting things (including various microorganisms) that may bring these products to marginal quality [93].

Although manufacturers (as they say) work hard to solve various problems to prevent contamination [94] (the current guideline says that 1.5 rodent hairs per 150 g of spirulina reflects quite good quality), it is hard to imagine efficient mechanical (non-chemical or physical) treatments that prevent birds, insects, amphibians, or rodents from infiltrating thousand-hectare open ponds. Although spirulina grows in basic media with high pH values, this biomass is susceptible to contamination from a wide range of pollutants and pathogens.

Apart from exogenous insects, birds, and rodents, cultivated cyanobacterial or microalgal biomass is a complex symbiotic system involving microalgae, (cyano)bacteria, and zooplankton. Even in closed bioreactors, while the target strain dominates, cross-contamination by biological pollutants is unavoidable. Therefore, there should be some ways to keep the pollution within certain limits so that the target cultivated species can reproduce and dominate the symbiotic system [95]. These include:

(1) The choice of species, namely stress-tolerant or enhanced in stability;
(2) Specific inhibitors and ambient conditions of biological pollution;
(3) Appropriate cultivation technology;
(4) Pollution monitoring;
(5) Quality control.

Detailed knowledge of possible and/or real contaminants is highly desirable. This may include metagenomics analysis of cultured strains [96] and identification of possible harmful organisms and substances produced by them [97]. In mixed culture populations, algal toxins can potentially contaminate products. Contamination and pollution are the most critical issues, since they can dramatically affect the quality of spirulina products, potentially resulting in foodborne diseases. In addition to health problems, contaminants may inhibit the growth rate and overall biomass production of spirulina [98].

Tight control of metal content is also desirable, since spirulina is known to accumulate considerable amounts of lead, copper, mercury, and other metals [99,100].

Spirulina is harvested through several rounds of filtration and then dried. Drying is an important aspect of high-quality biomass production. Various methods are utilized in the industry for drying spirulina. Freeze drying would improve overall product quality, but the expense is exorbitant. Usually, spray dryers are used in large-scale spirulina production; spirulina droplets are sprayed into the drying chamber to evaporate water. The resulting powder is exposed to 60 °C for a few seconds before falling to the bottom. The drying process uses no preservatives, additives, or stabilizers, and the powder is never irradiated [98]. This fast spray drying procedure guarantees the preservation of heat-sensitive nutrients, pigments, and enzymes. In line with good manufacturing practices, the product is not handled by human hands during harvesting, drying, and packaging.

General quality and safety standards for spirulina biomass have been described in comprehensive reviews [97,101]. To combat biological contaminations (bacteria, fungi, microalgae and their toxins, viruses, zooplankton) different approaches are used. They include filtration; chemical, antibiotic, or botanical pesticide (such as celangulin, azadirachtin, toosendanin) treatments; exposure to high temperature or pressure, or ozone; irradiation with electron beams or gamma-rays; and, finally—the most advanced to date—cold plasma treatment, an innovative non-thermal approach for food preservation and decontamination [97,102].

There has been a long discussion over whether drying reduces the amount and/or quality of bioactive compounds in spirulina. A comparative study of certain bioactive substances (flavonoids, phenolic acid, and antioxidant activity) found that fresh spirulina produces better results in bioactive components, both quantitatively and qualitatively, than dried samples. In terms of nutritional quality, the drying procedure at 40–50 °C has no noticeable effect when compared to fresh samples [103].

In addition to quality control, standardization of spirulina biomass parameters is critical. A study of 12 biomass samples obtained from producers in 10 countries found an important connection between FA content (primarily linoleic and γ-linolenic acids) and biological (immune-stimulating) activity. FA content varies by two times, while activity varies by 12.5. Quantitative methods have been developed for identifying FAs as potential chemical markers of immune-enhancing activity [104]. The large variation between samples emphasizes the importance of using standardization techniques to ensure consistency and accurate assessment of biomass characteristics for customers and future scientific research.

## 4. Genetic Modification of Spirulina: Achievements and Perspectives

The first reported attempt to construct a spirulina DNA library was conducted by Hiroyuki Kojima’s group at Osaka National Research Institute (Japan) with a method based on sonication and ligation with a TA vector [105,106]. For many years, the attempts to transform spirulina were unsuccessful. The persistent attempts to genetically modify spirulina resulted in transformation methods based on electroporation of linearized plasmid [107] or Tn5 transposon/transposase with cation liposome complexes [108]. Unfortunately, these relatively easy-to-use protocols have not been reproduced.

Only after a decade was an efficient and stable transformation method of spirulina reported [109]. During this time, it became clear that an effective DNA restriction mechanism outside or within the cells may be a barrier to the route of foreign DNA entering spirulina cells [110]. The restriction modification systems were characterized for spirulina, which consisted of Type-I (*Spl* I, *Spl* II, and *Spl* III [111]) and Type-II (*Apl* I [112]) restriction endonucleases, as well as many other candidates calculated on the basis of genome sequence analysis [113]. For transformation, electroporation and liposomes to protect foreign DNA have been applied together with the inhibitor of a type I restriction modification system [114].

An *Agrobacterium*-based system was also reported to transfer the reporter GFP:GUS and hygromycin resistance genes into the genome of spirulina [114].

Another strategy for transforming spirulina is to use its xenic culture with other bacterial species that produce inhibitors of various DNases [115]. Double homologous recombination, which is commonly employed to transform model cyanobacterial strains capable of natural transformation [115], was used to achieve stable, high-level production of an antibody against campylobacter. Following targeted integration of exogenous genes into the spirulina chromosome, encoded protein biopharmaceuticals accounted for up to 15% of total biomass, and require no purification prior to oral delivery. This dry biomass was stable without refrigeration, and it was protected during gastric transit when encapsulated in vegetarian capsules [114]. This simple recently published procedure (2022) has been cited a few times as a real breakthrough in biomedical science, but it has yet to be repeated.

An even simpler transformation approach has been proposed that does not require any specific inhibitor(s). This methodology relies on spirulina’s innate transformation capacity, double homologous recombination, and further antibiotic selection [116,117]. If it works, the lives of spirulina experts will become easier and brighter.

Spirulina genome editing may be achievable in the future. CRISPR and Cas systems use the self/alien discrimination principle to mediate protection against invading viruses and mobile genomic elements. An in silico analysis of the *Limnospira platensis* PCC 9108 genome identified six CRISPR arrays and five Cas gene clusters [118].

Furthermore, four complete sets of type I and thirteen type II restriction modification systems have been found, as well as several incomplete type I or II systems (for example, only methylase) and six type II systems produced by a single enzyme having both capabilities. The restriction enzyme genes include *Spl* I, *Sna*B I, *Hgi*C I, *Ava* I, *Ava* III, *Nsp* I; all of these activities had been experimentally proved [112].

It is worth mentioning that all cyanobacteria are characterized by the presence of so called *highly iterative palindrome-1* (HIP1), which is the octameric sequence, GCGATCGC. It appears exclusively and quite frequently in cyanobacteria: 7356 matches in the genome of *Synechococcus elongatus* PCC 6301 [119], or appearance in about half of *Synechocystis* sp. strain PCC 6803 genes [119,120]. The exceptions are unusual thylakoid-less *Gloeobacter* species, where this palindrome is absent [121]. HIP1 was first identified at the borders of a gene deletion event [118]. This sequence is, in fact, a recognition site for the restriction endonuclease *Sgf* I (*Asi*S I or *Rga* I, 5′-GCGAT^CGC-3′). Although these enzymes have not been found in cyanobacteria, sometimes they help to manipulate with cyanobacterial genes allowing deletions, insertions, etc.

## 5. Spirulinomics (Limnospiromics)

### 5.1. Genomics—Whole-Genome Sequencing

The complete genome of spirulina was not determined for a long time, mainly due to a large number of tandem repeats (TRs) and spirulina-specific repeats. Finally, in 2010, the draft genomes of several spirulina strains were resolved.

The *Arthrospira* (*Spirulina*) *platensis* NIES-39 genome is represented by a single circular chromosome of 6.78 Mb [121]. Annotation of this sequence revealed 6630 protein-coding genes, two sets of rRNA genes, and 40 tRNA genes. Of the protein-coding genes, 78% are identical to those found in other organisms; the remaining 22% are unknown. The genome contains 612 kb of group II introns, insertion sequences, and some repetitive elements. Group I introns are found in protein-coding regions. Numerous restriction modification systems have been identified. Unique gene composition traits were discovered, particularly in a large number of genes for adenylate cyclase and hemolysin-like Ca^2+^-binding proteins, as well as chemotaxis proteins. Comparative genomic analysis revealed filament-specific genes [121].

The genome of *Arthrospira*/*Spirulina* sp. PCC 8005 confirmed its highly repetitive nature, with more than 300 kb present as tandem sequences, and it contains four clustered CRISPRs, which may provide a cellular defense against phages and plasmids. The genome also contains at least 140 complete insertion elements (ISs) belonging to various families and eight copies of a putative genomic island [122].

The genome of A. platensis C1 (PCC 9438) appeared to be a little shorter (6.08 Mbp), existed in a single copy, and contained no plasmid DNA [123]. A total of 6153 open reading frames for putative proteins were predicted. Of these, 3757 were annotated as coding for proteins of known functions and 45 for RNA genes (6 for rRNA and 39 for tRNA). Highly interspersed repetitive sequences accounted for 9% of the whole genome.

*A. platensis* YZ, a spirulina species, was isolated from Chenghai Lake in Yunnan Province, China. Its circular chromosome is 6.62 Mb in size and contains 6784 protein-coding genes, 6711 of which have been annotated [124]. The genome sequence had two sets of rRNA genes and 39 tRNA genes, all of which were predicted to translate 19 distinct amino acids except for lysine. The latter is most likely hidden in a small, yet unsequenced portion of the genome. Otherwise, it is difficult to figure out how the organism handles itself without lysine.

Comparative analysis of the known spirulina genomes revealed that the unique DNA regions in Arthrospira sp. PCC 8005 and A. platensis C1 were markedly reduced relative to that in A. platensis YZ and A. platensis NIES-39. In silico hybridization identified 100 genes specific for A. platensis strain YZ, 82 of which encoded unknown proteins [124].

A draft genome of a cold-resistant spirulina strain obtained from Solenoye Lake in Siberia did not identify any unique genes related to its tolerance of low temperatures, even though that strain displayed the maximum growth index at 20 °C, compared to the 30–35 °C of Asian, African, and South American strains [125]. In general, Siberian strains did not differ from tropical strains in terms of the presence of stress response genes.

Despite numerous attempts at obtaining the entire genome sequence of spirulina, there were no closed genomes for a long time due to short-read sequencing technology, which resulted in multi-contig assemblages due to frequent extended repeats. In 2021, the *L. fusiformis* genome was sequenced with Oxford Nanopore technologies and assembled using ultra-long reads (>35 kb), followed by Illumina MiSeq short reads (San Diego, CA, USA). This resulted in a fully closed genome of 6.42 Mb, assembled as a single contig with no plasmid [126]. The DNA coding region accounted for 82.8% of the genome, with 5994 genes, including 5344 protein-coding genes, 51 RNA genes, two rRNA operons, and 41 tRNAs. A total of 29.6% of the genome was identified as repetitive DNA, containing 518 transposases, 12 recombinases, and seven CRISPR arrays. The genome contained the genes for 37 reverse transcriptases and eight phage annotations, suggesting viral associations with spirulina [127].

Another complete genome of the *L. platensis* strain NIES-39 (PacBio sequencing technology) was recently reported. It consisted of circular DNA of 6,818,916 bp (6.78 Mb in draft version [128]), which contained 6373 predicted protein-coding sequences (6630 in draft sequence), four rRNA genes (identical to draft sequence), and 48 tRNA genes (40 in draft sequence), including one gene for tRNA-Lys (locus_tag = “N39L_t00350”; https://www.ncbi.nlm.nih.gov/nuccore/AP026945; accessed on 16 July 2024) [129].

### 5.2. Transcriptomics

Due to high polysaccharide content, the isolation of nucleic acids from spirulina is complicated. At least RNA cannot be easily isolated with conventional liquid nitrogen grinding, or Trizol™ (Invitrogen, Waltham, MA, USA), or hot phenol extraction followed by lithium chloride precipitation [130]. It is also complicated because, in addition to new generation sequencing (NGS), there is a new generation of scientists who used to enjoy ready-to-go kits without any care about their contents and reaction mechanisms. Nevertheless, these difficulties can be overcome with “a little push” coupled “with a little luck” [[131,132,133]; https://www.youtube.com/watch?v=w99NAM1eMsg; accessed on 13 August 2024].

Spirulina transcriptome profiles were evaluated under various stress conditions, including ion starvation. Sulfur, primarily in the form of sulfates (SO_4_^2−^), has a considerable impact on the growth of cyanobacteria. Reduced sulfate is employed for protein (cysteine biosynthesis and subsequent transfer to methionine, glutathione) and coenzyme synthesis, whereas oxidized sulfur compounds are used for sulfolipid biosynthesis [134].

Sulfur deficiency led to an increase in total transcripts from ~6000 to ~7000 [135]. Annotated transcripts climbed from 4616 to 5250, whereas unannotated transcripts rose from 1407 to 1790. Sulfur depletion changed the expression patterns of genes implicated in multiple sulfur-dependent processes. Iron–sulfur cluster production was among the major genes that were downregulated due to the absence of sulfur. During sulfur depletion, genes for proteins involved in pathways such as translation, amino acid biosynthesis, protein folding, and rRNA binding were downregulated, whereas genes for proteins involved in carbohydrate metabolism, phosphor relay sensor kinase activity, integral membrane components, plasma membrane, thylakoid membrane, metal ion binding, and DNA repair were upregulated. Genes encoding proteins involved in transcription were upregulated, resulting in a greater number of transcripts in spirulina grown in a sulfur-depleted environment. However, genes for proteins involved in translation were downregulated, resulting in a decrease in total protein content. Thus, spirulina withstands sulfate stress by altering the expression of genes involved in sulfur metabolism and other sulfur-dependent pathways.

The effects of different salinity and nutrient concentration have also been studied on the transcriptome level [136]. The latter article contains amazing information: “In addition, polyunsaturated fatty acids (PUFA)—such as linolenic acid, stearidonic acid, and arachidonic acid—as well as macro and trace minerals, are abundant in *S. platensis*” [136]. Stearidonic acid (C18:4) cannot be synthesized by spirulina species, since it has only three FADs, while the synthesis of stearidonic acid requires four FADs (ω-3 FAD is missing in spirulina). Arachidonic acid (C20:4) should also be excluded from the list, since cyanobacteria do not carry genes for FA elongases, and the FA desaturation system in *Arthrospira*/*Limnospira* is still limited to trienoic FAs produced by Δ9-, Δ12-, and Δ6-FADs (Figure 3).

The study [136] concluded that under nutritional and/or salt stress, spirulina responded by modifying gene expression involved in growth regulation and ATP generation. The problem with such an experimental setup is that they all use transcriptomics to assess the events that occurred several hours (or even days) after adaption to various stressors. The measurement of gene transcript levels within the initial few minutes or hours of stress treatment is critical for understanding the stress-regulatory mechanisms. Prolonged acclimation or adaptation of cells causes severe metabolic alterations, after all necessary proteins or regulatory RNAs have been synthesized and the associated metabolites have been produced. At this time, the analysis of transcripts is not very instructive, or, at least, it does not provide information about changes in metabolic pathways at the transcription level.

In view of this, the comprehensive investigation of physiological changes and whole-genome expression is preferable. An example was the testing of spirulina reactions to high temperature, a shift from 35 °C to 42 °C for 2, 8, and 24 h. The results of the expression profiling in response to heat stress revealed two distinct phases for the responses. The first was the immediate response phase, in which the transcript levels of genes involved in different mechanisms, including genes for heat shock proteins, signal transduction, carbon and nitrogen metabolism; inorganic ion transporters were either transiently induced or repressed by heat. In the second, long-term response phase, both the induction and repression of genes with important roles in translation and photosynthesis have been observed [137].

### 5.3. Proteomics

Temperature responses of spirulina have also been assessed by proteomic approaches [138,139]. On the protein level, low-temperature stress was tightly linked with oxidative stress as well as photosynthesis. However, no specific responses have been revealed under high-temperature stress. Indeed, the induction of so-called heat stress proteins (HSPs) in cyanobacteria appeared to be non-specific. HSPs are induced by heat, salt, osmotic, acid, UV, oxidative stresses, and redox changes; all resulted in transcriptional activation of major HSPs and, as a consequence, accumulation of the corresponding proteins (17, 60, 70, 90 kDa) [140,141]. Temperature stress affected proteins involved in nitrogen and ammonia assimilation and highlighted the cross-talk of signaling pathways. Several sensory histidine kinases (Hiks), e.g., Hik14, Hik26, and Hik28, have potential interactions with differentially expressed proteins under both high- and low-temperature stresses [139].

Protein expression profiles have also been compared in spirulina exposed to different temperature (15 °C, 35 °C, and 45 °C) [142]. A total of 122 proteins having a significant differential expression response to temperature have been determined and assigned to 14 functional groups. These proteins are mainly involved in post-translational modifications, protein turnover, energy metabolism (photosynthesis, respiratory electron transport), translation (ribosomal structure and biogenesis), carbohydrate and amino acid transport and metabolism, cell envelope biogenesis, coenzyme metabolism, and signal transduction.

An isobaric tag for relative and absolute quantitation (iTRAQ)-based quantitative proteomics approach identified a total of 3782 proteins, of which 1062 showed differential expression under cold stress [143]. Upregulated differentially expressed proteins were assigned to carbohydrate and amino acid metabolism. The downregulated expression of proteins involved in glycolysis, TCA cycle, pentose phosphate pathway, photosynthesis, and translation was associated with reduced energy consumption.

Apart from stress-regulated proteins, bioactive proteins and peptides attract the attention of investigators, since spirulina biomass fractions are known as antioxidant, anticancer, and antihypertensive agents. Proteomics and bioinformatics approaches have been combined to search for novel protein sources for bioactive peptides. The 593 high-abundance proteins of spirulina were subjected to in silico digestion by trypsin, pepsin, and chymotrypsin, giving 78, 99, and 96 bioactive peptides, respectively [144]. After the extraction of ordinary products from spirulina, *C*-phycocyanin and lipids, the remaining biomass contains proteins with potentially bioactive peptides. Those proteins, digested with papain, alcalase, trypsin, Protamex 1.6, or Alcalase 2.4 L, resulted in hydrolyzed peptides that possessed antioxidant activity. LC-MS/MS analysis revealed 230 peptides with various biological activities derived from 108 spirulina proteins [145]. Protease K protein hydrolyzation and reverse phase HPLC separation followed by LC-MS/MS revealed 30 peptides with angiotensin I-converting enzyme (ACE)-inhibitory activities. Further investigation identified peptides, TVLYEH and LQAGGLF (both of 0.7 kDa), with the highest ACE competitive inhibitory activity [127]. Phycobiliprotein-derived bioactive peptide, IRDLDYY (1 kDa), characterized by high-affinity binding to ACE, also possessed ACE inhibitory activity [146]. Another spirulina peptide revealed by proteomics, GIVAGDVTPI (1 kDa), improved endothelial vasorelaxation, and exerted an antihypertensive action through a NO-dependent mechanism [147]. Thus, spirulina protein hydrolysate may be used as nutritional supplement for blood pressure control.

In fact, it seems that whatever pharmaceutical drug one wants to get from spirulina, he/she can get it, including antioxidant, antidiabetic, immunomodulatory, antihypertensive, and antihyperlipidemic agents [148,149,150,151,152].

If spirulina fails, even a wider range of bioactive chemicals can be isolated from the marine cyanobacterium, *Moorea producens* [153] (formerly—*Lyngbya majuscula*) or some other cyanobacterial species [154]. Some health-protective properties of spirulina are reflected in systematic reviews and meta-analyses of randomized clinical trials (Table 2).

Proteomic methods (LC-ESI-FTMS/MS) have been also applied in order to find highly conserved peptides as markers of spirulina occurrence in foodstuffs like pasta, crackers, bread, etc. Initially, putative markers have been chosen by in silico protein digestion, and, finally, three peptides originating from *C*-phycocyanin beta subunit were designated as qualifiers (ETYLALGTPGSSVAVGVGK and YVTYAVFAGDASVLEDR, both of 1.9 kDa) and quantifier (ITSNASTIVSNAAR of 1.4 kDa) marker peptides and used for validation of commercial foodstuffs [155].

**Table 2:** Health benefits of spirulina as reported in systematic reviews *.

Effects	Targets	References
Antiviral	Immunodeficiency virus (HIV)	[156]
	Hepatitis C virus (HCV)	[156]
	COVID (SARS-CoV-2)	[157]
Antioxidant	Free radicals, lipid peroxidation	[158]
Anti-inflammatory	NO-synthase, tumor necrosis factor (TNF)-α and interleukin-6	[157,158,159]
Anticancer	Inhibition of the proliferation of tumor cells, triggering cell cycle arrest, and induction of apoptosis via different signaling pathways	[160]
Antidiabetic	Improving fasting blood sugar, total cholesterol, triglycerides, increasing the high-density lipoprotein cholesterol	[161,162]
Antimicrobial	Inhibition of bacterial growth via antimicrobial peptides, phenolic compounds (no exact mechanism reported)	[163]
Antitoxic	Protection from poisonings from arsenic, cadmium, carbon tetrachloride, deltamethrin, fluoride, hexachlorocyclohexane, iron, lead, mercury (detoxification by absorbtion)	[164]
Hypertension	Lowers systolic and diastolic blood pressure (no mechanism reported)	[165]
Obesity	Reduces body weight (no mechanism reported)	[166]
Hepatoprotection	Improvement of sonographic liver parameters (no mechanism reported)	[167]

* The amount of dry tableted or capsuled *Spirulina* ranged from 1 to 8 g per day, and intervention durations ranged from 2 to 12 weeks.

### 5.4. Metabolomics

The metabolome is the complete set of metabolites, or non-genetically encoded substrates, intermediates, and products of metabolic pathways associated with a cell. Given the increasing requirement to quantitatively and qualitatively estimate the bioactive compounds and understand the complex formulations of natural medicinal products, metabolomics has emerged as an important technology to cell-wide measurements of primary and secondary metabolites and metabolite fluxes. Metabolomics already became a tool for drug discovery and development, metabolic engineering, water and food quality control [168,169]. The metabolomics protocols usually combine gas chromatography coupled to mass spectrometry (GC–MS), ultra-high performance liquid chromatography coupled with high-resolution tandem mass spectrometry (UPLC-HRMS/MS), and ultraviolet–visible (UV/Vis) spectrophotometry [170].

The multiple pharmaceutical applications of spirulina metabolites and their impact on various health disorders would certainly appeal to metabolomics approach for fractionation and characterization of the spirulina biomass [171,172]. Surprisingly, instead of great hopes and expectations, the metabolomics data on spirulina bioactive compounds is still missing in open scientific press.

Instead, metabolomics methods are widely applied for quality control of crude spirulina products [173]. For example, drying methods—freeze and sun drying, air, infrared, and vacuum (all performed at 40 °C or 75 °C) drying have been compared using reversed-phase liquid chromatography separation coupled with high-resolution tandem mass spectrometry with electrospray ionization (RP-LC-ESI-Orbitrap HRMS/MS). Totally, 316 metabolites have been identified in aqueous and ethanolic extracts. Using freeze drying (most expensive and most effective method) as a control, the most protective method appeared to be air drying at 40 °C. Vacuum drying at both 40 and 75 °C caused the most intense degradation of valuable spirulina metabolites [174].

So far, only few studies have been focused on identification of bioactive compounds of spirulina, which participate in skin care [175] or wound healing [176]. Some spirulina phytochemicals are particularly efficient in wound healing since they enhance wound closure by increasing the growth factors involved in the healing process, controlling collagen synthesis, and promoting its deposition in the wound bed. Thirteen compounds have been identified, including phenolic acids, fatty acids, alcohols, and nucleoside derivatives, which may affect wound healing [177]. Among phenolic acids, cinnamic acid, 4-hydroxybenzoic acid, salicylic acid, and benzoic acid have been identified. A monoterpenoid hydroxy lactone, loliolide, has been identified, which reduces ROS-induced apoptosis. Pantothenic acid (vitamin B5) that facilitates fibroblast proliferation was also found. Azelaic acid eliminates inflammation, exfoliates dead cells, has an antiseptic effect and aids in the removal of ROS. Ophthalmic acid is a precursor of glutathione, which was reported to reduce oxidative stress [178]. In addition to skin care compounds, a bonus 12,13-dihydroxy-9Z-octadecenoic acid (12,13-diHOME) has been found in spirulina extract—a lipokine that increases skeletal muscle FAs uptake and prevents obesity [179].

## 6. Spirulina in Space

If spirulina already has the proud name of Superfood on Earth, why not think about conquering the universe? The idea to employ microalgae in space vehicles and orbital stations for scavenging (fixation) of CO_2_ and production of oxygen, as well as for foods and recycling, came even before the launch of Earth’s first cosmonaut, Yuri Gagarin [180]. It was thought that unicellular algae will become a good diet of cosmonauts/astronauts because they consume a minimum of space due to their ability to grow in closed bioreactors. *Chlorella*, as a representative of eukaryotic microalga, became a passenger of the second Soviet satellite in 1960. It safely came back to Earth, and the results of the experiments on its survival and some characteristics had been reported in 1962 [181]. *Chlorella* appeared not the best choice for everyday human consumption because of its very hard cell envelope. It is more suitable for cattle with three sectional stomach adapted to digest huge amounts of grass cellulose. The bravest astronauts could consume substantial amounts chlorella biomass, but suffered from flatulence (which is not comfortable in a limited closed space with no chance to open a ventilation hatch). In that sense, spirulina is much more suitable microorganism with more appropriate human-oriented nutritional properties.

The investigations of spirulina grown in bioreactors installed on board of the International space station (ISS) and on the ground revealed no significant difference in the oxygen production and biomass accumulation. Although, due to technical difficulties, a definitive conclusion on the influence or lack of influence of the microgravity on the spirulina growth could not be reached, it appeared that the terrestrial bioreactor growth model is applicable for representing zero-gravity conditions [182]. It has been proposed that microalga-based life support systems are cost-effective for long-term space missions [183,184,185].

Indeed, one of the challenges of long-duration space missions is the limited life support resources that may be carried onboard. These missions would benefit from processes or systems capable of performing long-term closed-loop life support tasks such as oxygen regeneration from CO_2_, supplemental food provision, and water remediation. In scenarios of long-term human planetary habitation, life support systems that may employ local resources (for example, CO_2_ in Mars’ atmosphere) may considerably improve mission success [183]. These processes have the potential to drastically alter space system design and configuration, improve mission flexibility, increase astronaut life support on-site independence, and pave the path for novel methods of producing life support materials in space. The successful completion of manned missions to Mars is one of the most significant problems that humanity will face in the future. In this regard, the idea of growing spirulina to create food for astronauts on Mars has been examined. The trials were conducted in a system capable of simulating microgravity and an inner atmosphere similar to Martian conditions (−27 °C, 0.8 kPa, 100% CO_2_). The results showed that many cyanobacterial strains of *Anabaena cylindrica*, *Chroococcidiopsis*, *Gloeocapsa*, *Phormidium*, and *Leptolyngbya* could survive several days under Mars-simulated conditions with a regolith simulant as a growth substrate [184]. Growing spirulina in photobioreactors simulating Martian dome hosting (growth medium containing a mixture of Mars regolith leachate and astronaut urine simulants) revealed that spirulina could grow with a higher productivity and growth rate than on Earth. Given the obtained productivity and astronauts’ requirements, a culture of around 15 m^3^ within pressurized domes would be sufficient to supply the protein demands of six crew members [185].

The most promising alga-based life support systems include phototrophic microorganisms that can be used to create carbon, oxygen, and nitrogen cycles within a single system, allowing for a reduction in the level of resupplied and/or stored resources, as well as the total crewed expedition cost. However, there are still a number of issues that require further investigation, the most important of which is long-term large-scale microorganism cultivation under the influence of significant stress factors such as cosmic radiation, microgravity/weightlessness, low atmospheric pressure, and high CO_2_ concentrations [185].

Currently, the ISS’s operating life support system, which uses physicochemical processes, can only recycle a portion of the water and recover a fraction of the oxygen required by the crew. The present system’s failure to produce enough food demands regular resupply flights from Earth, rendering it unsuitable for long space journeys (e.g., a crewed Mars mission) due to the restricted cargo capacity of current rockets. Thus, the life support system in entirely isolated environments must be bio-regenerative. The latter is defined as “closing the loop” in the regeneration of air, food, water, and other components that support human existence via biological systems (especially those involving autotrophs).

The closed-loop life support system reduces the requirement for resupply by recovering valuable resources from waste. The ability of biological systems to provide the majority of the necessary life support components will be the primary means of assuring crew members’ long-term survival in space. Physical–chemical systems will only be employed as a backup or redundancy. Microalgae were found to have benefits over plants in terms of edible biomass productivity, cultivation period, and growth requirements (water and illumination).

Terrestrial and space studies have demonstrated that several microalgal and cyanobacterial species may thrive on human-derived wastewaters, space-derived nutrients (i.e., Martian regolith), and CO_2_, indicating their potential for in situ resource utilization. Nonetheless, cultivating microalgae under true space conditions presents numerous obstacles that necessitate additional research before inclusion in bio-regenerative life support systems for long-duration space flights [186].

## 7. Conclusions

Recent advances in omics technology have enabled rapid progress in the study of spirulina, an ancient edible cyanobacteria. Phylogenetic investigations and bioinformatics approaches discovered numerous lines of spirulina species, which were then divided into three distinct genera: *Spirulina*, *Arthrospira*, and *Limnospira*. The latter consists of only one strain, *Limnospira platensis*, with a number of substrains, which are industrially grown and sold under the commercial name of spirulina. Whole-genome sequencing identified several genes involved with stress tolerance, restriction modification mechanisms, and metabolite production, shedding light on spirulina physiology. Genomics, proteomics, and metabolomics demonstrated the absence of hazardous compounds in spirulina cells, confirming the safety of this biomass as a food product. Spirulina never produced ω3 FAs or FA carbon chains longer than C18 atoms. Recent advancements in spirulina transformation methods enabled the creation pharmaceutical compounds, which can be eaten directly with spirulina biomass, whether fresh or dried. Genetic transformation may also allow the synthesis of ω3 FAs (which have never been synthesized by natural species of *Spirulina*, *Arthrospira*, or *Limnospira*). Spirulina’s enrichment with inherent biologically active chemicals makes it a possible source of natural pharmaceuticals used to treat a variety of health issues. Spirulina is also a prospective component of both terrestrial and space-based life support systems. The existence of spirulina everywhere means that it benefits human lives and drives further research and discoveries.

## Figures and Tables

**Table 1:** The appearance of commonly known substances in spirulina.

Substance	Relative Quantities	References
Allophycocyanin	Up to 47% of total protein	[34]
Carotenoids (β-carotene, zeaxanthin, cryptoxanthin)	Up to 2% (d/w *)	[71]
Chlorophyll *a*	Up to 2% (d/w *)	[31]
Chlorophyll *b*	0	[31]
Palmitic acid	42–47% of total FAs	[53,55]
Oleic acid	2–5% of total FAs	[53,55]
Linoleic acids	15–35% of total FAs	[61]
γ-Linolenic acid (GLA)	10–50% of total FAs	[63,64,65]
ω3-FAs	0	[72]
α-Linolenic acid (ALA)	0	[72]
Eisosapentaenoic acid (EPA)	0	[72]
Docosahexaenoic acid (DHA)	0	[72]
Triacylglycerides (TAGs)	0	[73]

* On a dry weight basis (d/w).

## Data Availability

No new data were created or analyzed in this study. Data sharing is not applicable to this article.
